# Supplementary material for: Repo-Man/PP1 regulates heterochromatin formation in interphase
Source: Nat Commun. 2017 Jan 16;8:14048. doi: 10.1038/ncomms14048 (PMC5241828; doi:10.1038/ncomms14048)
Supplement: Supplementary Information — Supplementary Figures 1-6 and Supplementary Tables 1-2 [file ncomms14048-s1.pdf]

Supplementary Figures

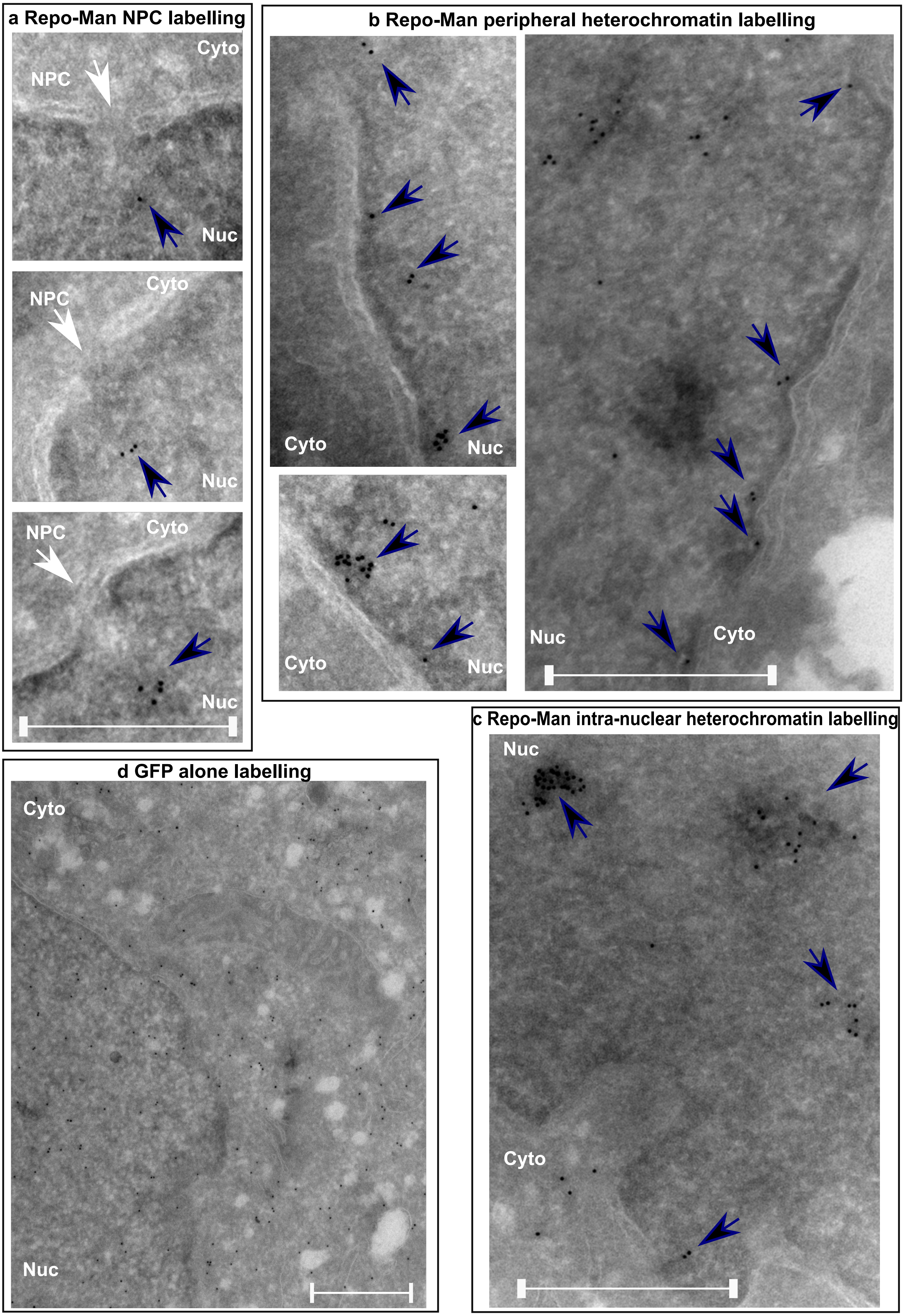

Supplementary Figure 1: EM labelling of Repo-Man and GFP cell lines.
HeLa cells stably expressing GFP:Repo-Man^{NTerm} were stained for GFP, immunogold labelled and visualised by EM. The fused protein localises adjacent to the NPC (a) and contigously with peripheral heterochromatin (b). Accumulation of Repo-Man is also seen at dense chromatin regions in the nuclear interior (c). Cells expressing GFP alone show widespread staining (d). bar = 500nm.

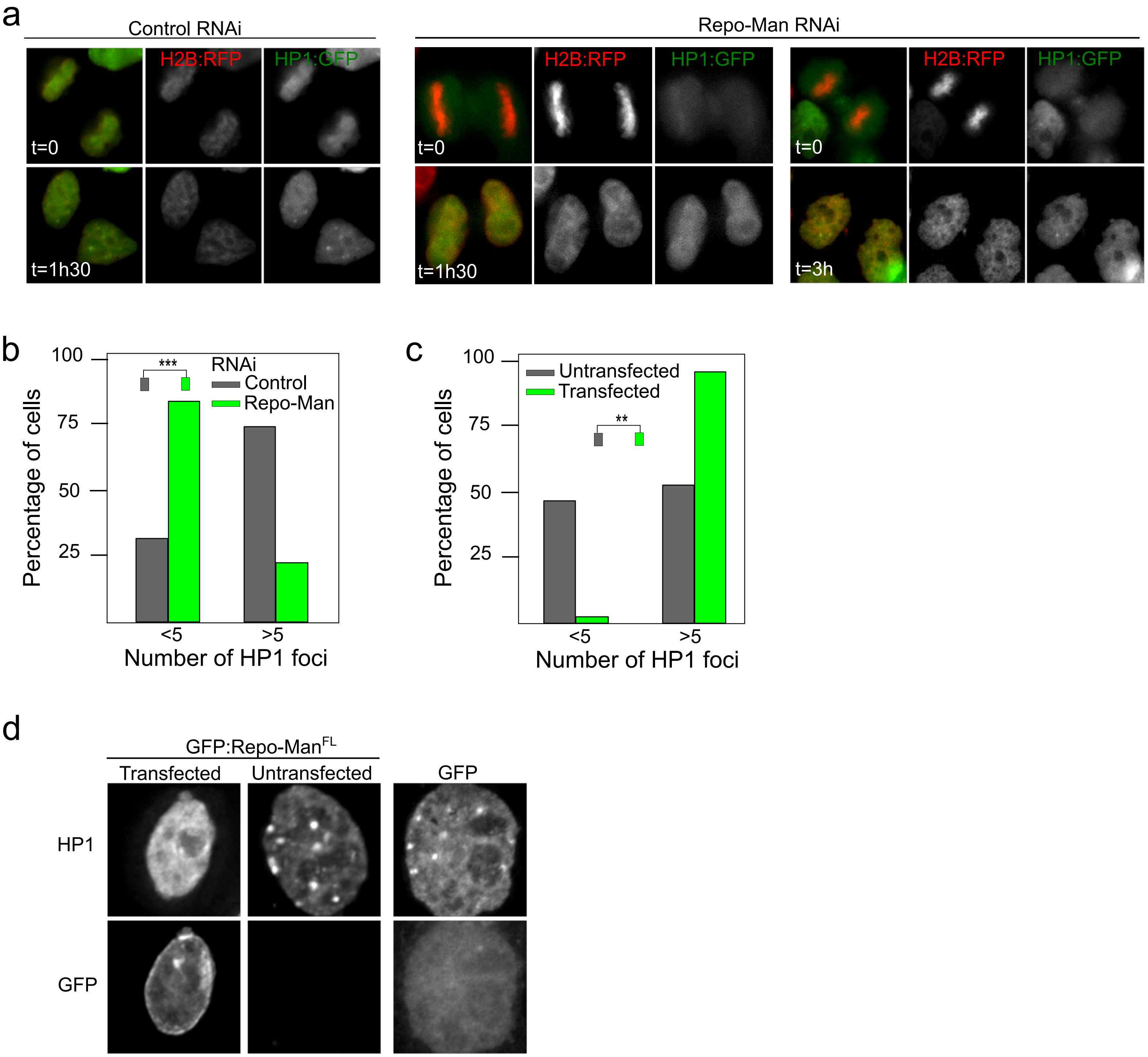

Supplementary Figure 2: HP1 is disrupted from mitotic exit and depends on Repo-Man levels.
(a) HeLa stable cells expressing RFP:H2B and transfected with GFP:HP1 were followed throughout mitosis upon control and Repo-Man RNAi. HP1 foci were present 1h30 after anaphase in control RNAi but absent after 1h30 and even 3h in Repo-Man RNAi. (b) Quantification of Repo-Man foci after cells passed through mitosis as described in (a). (c) GFP:Repo-Man full length was transiently expressed in HeLa and cells stained for HP1. HP1 foci were counted in transfected and untransfected cells. (d) Examples of HP1 staining in cells transfected or untransfected with GFP:Repo-Man full length. As a control, cells transfected with GFP alone were also stained for HP1. Chi-square test was applied. ** p <0.01; *** p<0.001.

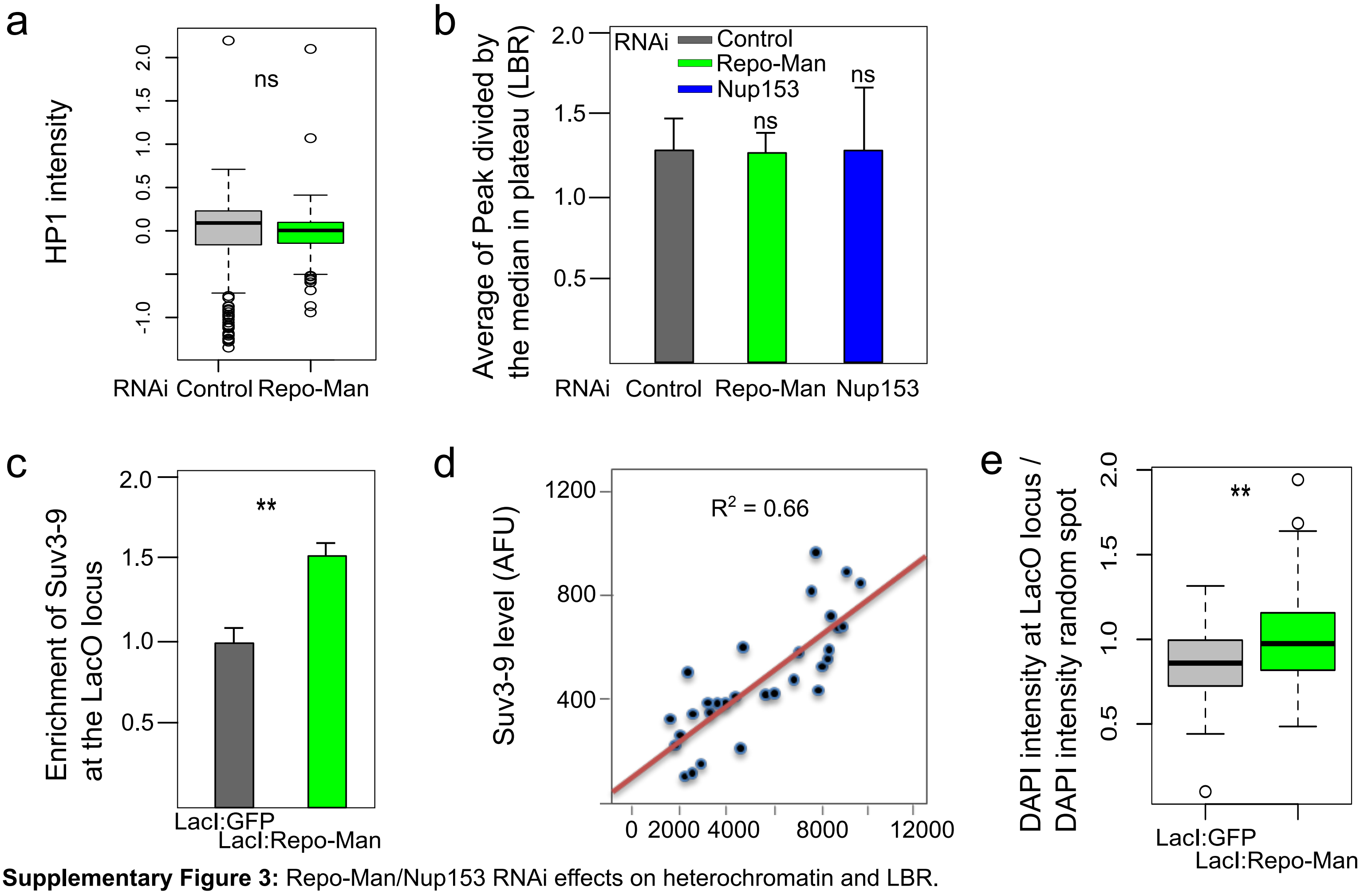

Supplementary Figure 3: Repo-Man/Nup153 RNAi effects on heterochromatin and LBR.
(a) Signal intensity of HP1 alpha foci after immunostaining of HeLa cells depleted for Repo-Man (green) and control oligo (grey).
(b) LBR enrichment at the periphery after immunostaining of HeLa cells depleted for Repo-Man (green), Nup153 (blue) and control oligo (grey). (c) Measurement of Suv3-9 signal intensity in GFP:LacI:Repo-Man or GFP:LacI at the LacO array (stars indicate t-test ** p<0.01). (d) Correlation between GFP:LacI:Repo-Man enrichment at the LacO array and the levels of Suv39 with linear regression.
(e) Measurement of DAPI signal intensity in the LacO array when DT40 cells are transfected with either LacI:GFP or GFP:LacI:Repo-Man (Mann-Whitney test was applied. ** p <0.01).

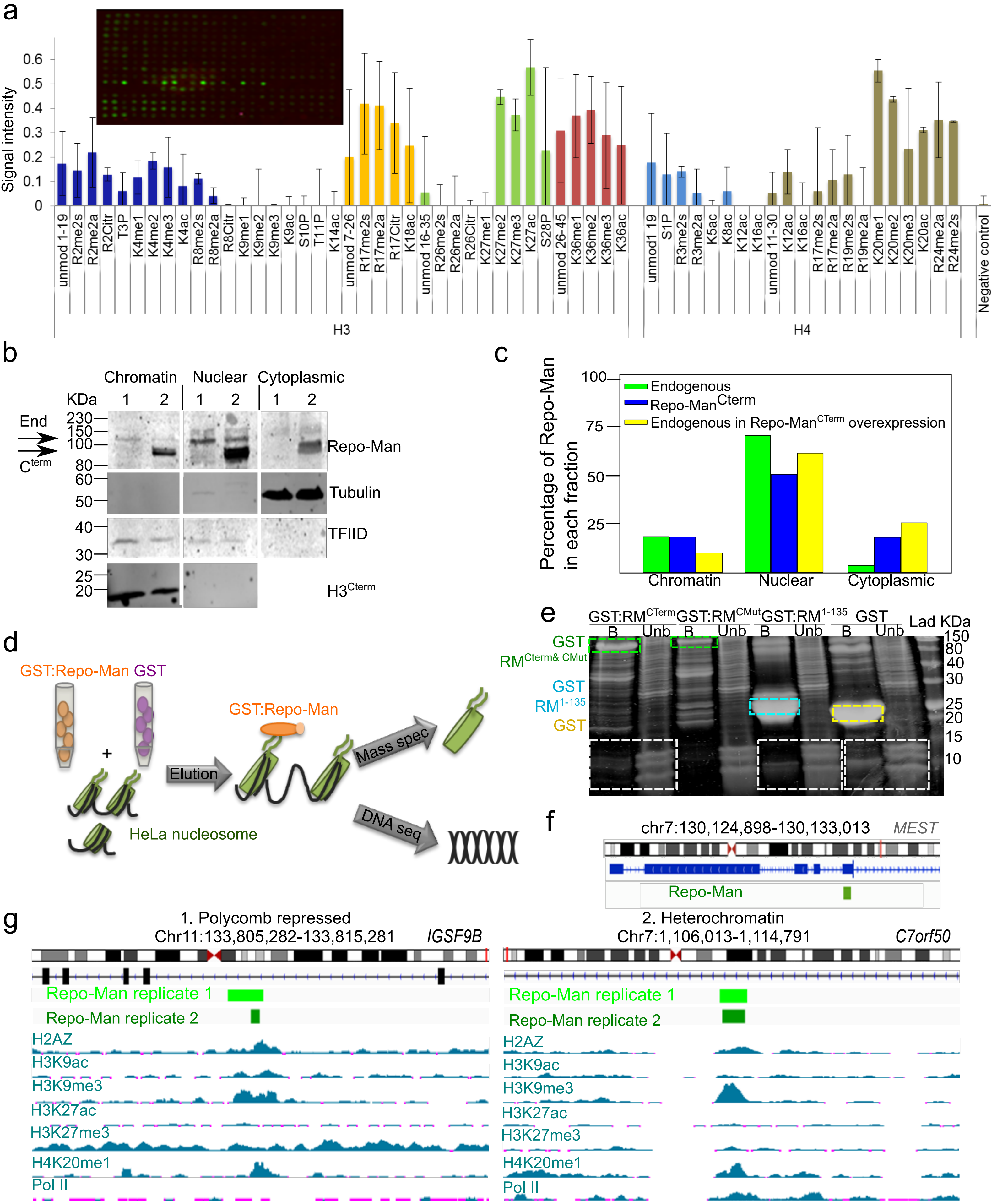

Supplementary Figure 4: Repo-Man interactions with chromatin.

(a) Recombinant GST tagged Repo-Man^{CTerm} or GST alone were incubated in a histone peptide array. The signal was detected with an anti-GST antibody and quantified with LICOR across two arrays (Fig. 4a). (b) Chromatin, nuclear and cytoplasmic extracts from HeLa cells expressing GFP (1) or GFP:Repo-Man^{CTerm} (2). TFIID, H3^{CTerm} and Tubulin antibodies were used as controls of nuclear, chromatin and cytoplasm fractions, respectively. Repo-Man antibody was used to detect endogenous and overexpressed RM. (c) Quantification of RM as in (b). (d) Overview of the TAG-proteogenomics. Recombinant GST:Repo-Man^{CTerm} or GST alone were incubated with nucleosomes extracted from HeLa. After pull-down, samples were separated by SDS-page and the histone fraction was analysed by Mass Spectrometry and the DNA extracted and sequenced. (e) White bars correspond to crops in Fig.4e. Coloured bars the expression of GST:RM^{CTerm}, GST:RM$^{1-135}$ and GST alone, correspondently. (f) Repo-Man binding across MEST gene. (g) RM genome hits on a polycomb repressed gene, *IGSF9b*, and on a heterocrhomatin gene, *C7orf50*, classified as such by ChromHMM (Fig. 5e).

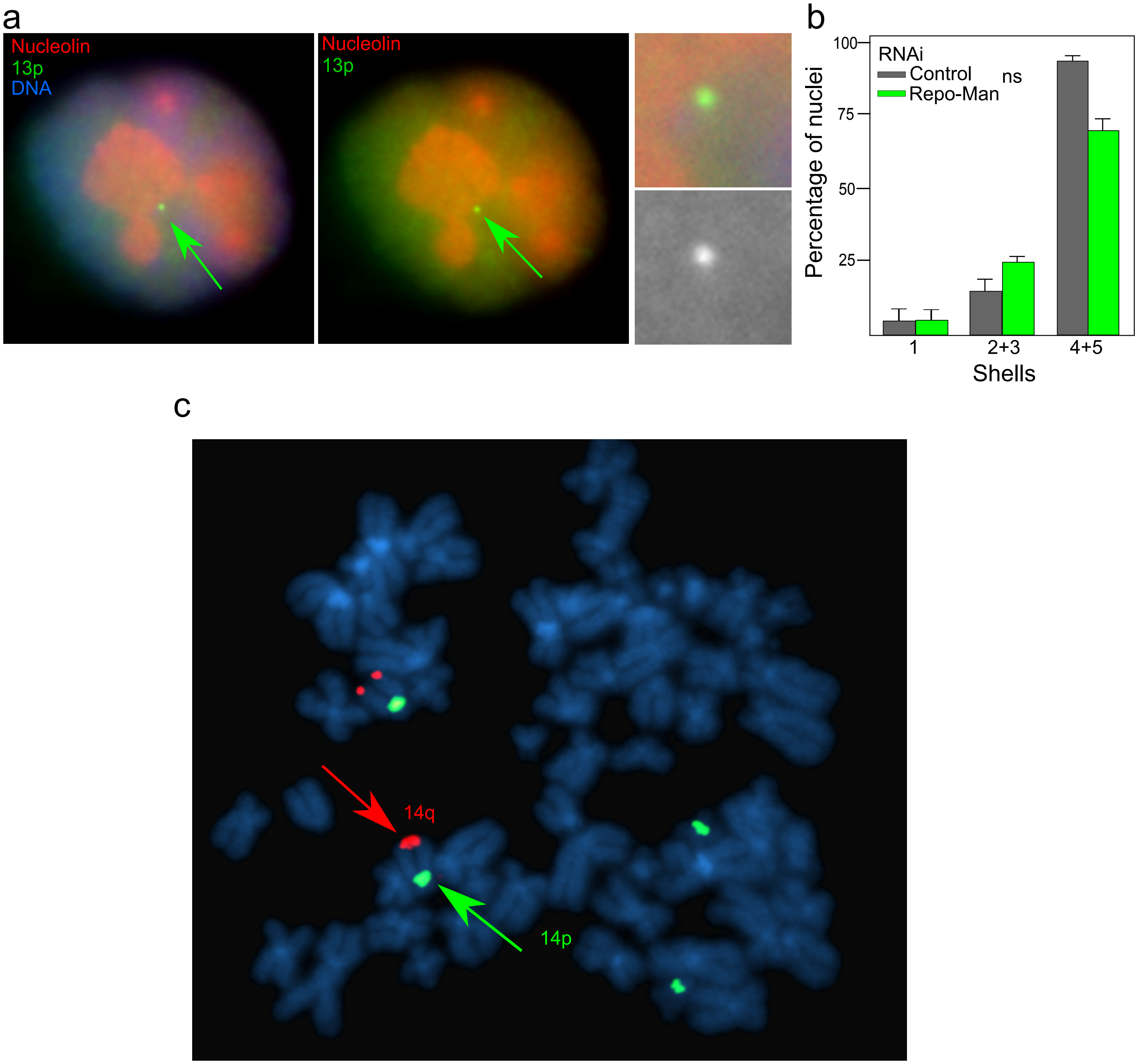

Supplementary Figure 5: Repo-Man organises the nuclear position of specific chromosome regions. (a) HT1080 cells containing a LacO array inserted at chromosome 13p and expressing GFP:LacI were fixed and stained for Nucleolin. (b) Position of the chr13p measured after RNAi with control (grey) or Repo-Man (green) oligos. (c) FISH on HeLa metaphase spreads with probe CTC-820M16 (red signal). The probe maps to the subtelomeric region of chromosome 14 (red arrow), as confirmed by the hybridization of the 22-14 alpha satellite probe (green signals), which labels the centromeres of chromosome 14 (green arrow) and 22.

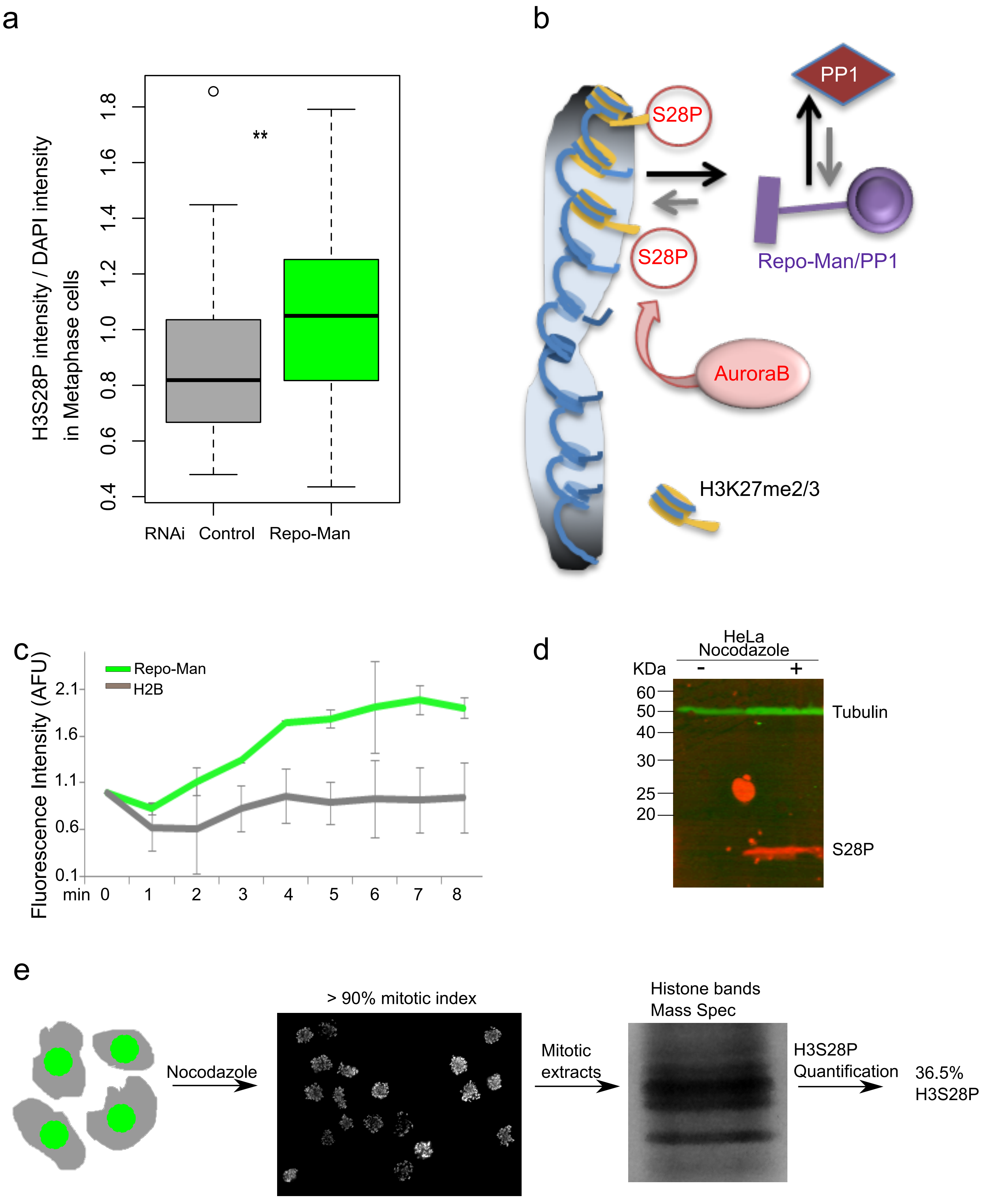

Supplementary Figure 6: Repo-Man dephosphorylates S28 in mitosis.
(a) Intensity of H3S28P signal in mitotic HeLa cells after control or Repo-Man RNAi (Mann-Whitney test, ** $p < 0.01$).
(b) Repo-Man/PP1 complex regulation in mitosis and its dynamic binding to chromatin maintains the correct H3S28P levels in mitosis.
(c) Repo-Man and Histone H2B levels on chromosomes after mitotic exit. (d) HeLa cells were treated with nocodazole overnight to induce mitotic entry. Western blot using Serine 28 phosphorylation antibody and tubulin as a control. (e) Cells were treated with nocodazole overnight to induce mitosis (90% mitotic index) and histones from mitotic extracts were sent to Mass Spectrometry, as example here, to evaluate H3S28P content (36.5%).

Supplementary Tables

Supplementary Table 1: Antibodies used in this study

ANTIBODY	SOURCE	DILUTION
Repo-Man	Protein Tech 17701-1-AP	1:250 (WB)
Repo-Man	In house	Vagnarelli et al. 2011 1:300 (IF)
H3K9me3	Active Motif 39161	1:1000 (WB) / 1:500 (IF)
H3K9me2	Active Motif 39240	1:500 (IF)
H3K27me2/3	Active Motif 39535	1:1000 (WB) / 1:500 (IF)
H3K9ac	Abcam ab10812	1:500 (IF)
H3S28P	Abcam ab10543	1:1000 (IF)
H3S28P	Novus Biologicals (NB21-1188)	1:500 (WB)
H3K9me3	Abcam ab71999	1:100 (IF)
H3-Cterm	Active Motif 39163	1:1000 (WB)
LBR	Abcam ab32535	1:200 (IF)
Suv39	Active Motif 39785	1:200 (IF)
HP1	Millipore MAB3584	1:200 (IF)
Nup153	Abcam ab24700	1:300 (IF)
c-Myc	Santa Cruz sc-40	1:200 (peptide array)
GST	Thermo CAB-4169	1:1000 (WB/peptide array)
TFIID	Santa Cruz sc-225	1:200 (WB)
GFP	Roche 11814460001	1:1000 (IF)
alpha-Tubulin	Sigma B512	1:1000 (IF) 1:3000 (WB)

Supplementary Table 2: qPCR primers used in this study

Primer	Sequence (5' to 3')
Expression	
GAPDH F	ACCACAGTCCATGCCATCAC
GAPDH R	TCCACCACCCTGTTGCTGTA
SLC6A19 F	CTCCAGCTACAACTCTGTGCAC
SLC6A19 R	CCAATGACGGAGTAGACCACGA
ADCY2 F	AATCAGGTGGCGATTCTGCGTG
ADCY2 R	AGTTTACCCGCAGGAACACGGA
GPR133 F	AAGACTCGGTTGCTGCTGTT
GPR133 R	GTCGGGCTCTCCACTCATTA
SLC6A18 F	GATGACGTAGGGGATGAGGA
SLC6A18 R	TACCTCCTGAGCTGCATTGG
PPP2R2C F	CCGTAAGGTCCTCCATGTTG
PPP2R2C R	CTACATGTCGGCGGATGAC
Repo-Man F	GAGGCAGGAAAAGAGTCCGAGA
Repo-Man R	CTCCGACGTTTGGAGGACAACA
ChIP	
MYT1 F	CCGTCAAGTCCCATTTTGGA
MYT1 R	AGTTCAGGAGAGAAGTTGCG
SLC6A19 F	ACGCATGCTTTCGTTCTTCT
SLC6A19 R	GAAACTGGAGCTTCCTGCAC
ADCY2 F	ACCGGCGGTTAAGACTTTTT
ADCY2 R	ACTCACGGAAAGCCTCAAGA
GPR133 F	GGGCAGCCATGTGTTAGAAT
GPR133 R	TAGTATCGGCCCCTCTGTTG
IGSF9B F	CCTGAAGCTCAGAACCCAAG
IGSF9B R	CTGATAGAGATGGCGGTGGT
PPP2R2C F	TGATAAGGGCCCAATAGCTG
PPP2R2C R	AAATTCCCCAAGATGCAACA
